# CAPON and Schizophrenia—Does Size Matter?

**DOI:** 10.1371/journal.pmed.0020329

**Published:** 2005-09-13

Schizophrenia and bipolar disease are complex diseases, with multiple genes and environmental factors thought to be responsible for their manifestation. Many reports have implicated changes in certain regions of the human genome in schizophrenia. An area on Chromosome 1 has been associated with the disease in different studies and populations. Linda Brzustowicz and colleagues had previously described association of several single nucleotide polymorphisms (SNPs) within a gene called *CAPON* (for carboxyl-terminal PDZ ligand of neuronal nitric oxide synthase) with schizophrenia in a set of Canadian families. A separate study in a Chinese population found an association between schizophrenia and a separate group of SNPs within *CAPON*. *CAPON* is an attractive candidate for a “schizophrenia gene”: CAPON was first identified as a protein binding to neuronal nitric oxide synthase (nNOS), and indirect evidence suggests that it might be linked to the regulation of glutamate neurotransmission. However, so far, no coding sequence mutations in CAPON have been found in patients with schizophrenia.

Brzustowicz and colleagues now report results from a study of CAPON expression in postmortem brain samples from patients with schizophrenia, from patients with bipolar disorder, and from control individuals without psychiatric illness. Initially screening a human fetal brain cDNA library for potential alternative splice forms of CAPON, they found, in addition to the predicted full-length transcript, a shorter isoform that consists of the last two exons of the gene. They also confirmed that both long and short versions of the protein are present in human brain. (The short isoform would still be able to bind nNOS and possibly disrupt its interaction with other proteins.)

The authors then examined *CAPON* mRNA expression in postmortem brain samples from 35 patients with schizophrenia, from 35 patients with bipolar disorder, and from 35 controls. They looked for transcripts encoding the long and short forms and also compared expression levels (relative to beta-actin) across the diagnostic groups. In the dorsolateral prefrontal cortex (thought to be involved in schizophrenia and the only area for which samples were available), expression levels of the long isoform did not differ between patient and control samples. However, the short isoform was expressed at higher levels in the patients with mental illness than in the controls. They also analyzed DNA from these individuals at three SNPs associated with schizophrenia and found, for each SNP, that the group of individuals who carried one or two copies of the schizophrenia-associated allele had overall higher levels of the short form transcript than those homozygous for the non-associated *CAPON* allele. They saw no differences in levels of the full-length transcript between the groups with different SNP genotypes.

These are intriguing but preliminary results. Getting reliable results from studies on postmortem samples is extremely difficult because of numerous confounding factors. Brzustowicz and colleagues tried to control for some of them (such as sex of the individual, substance abuse by the individual, and storage time of the sample), but others are impossible to determine or control for. Moreover, this particular collection of samples had only tissue from the dorsolateral prefrontal cortex available for study. Sample preparation was geared toward high-quality RNA for expression studies and not suitable for parallel protein analysis. Future studies, with at least some of them in animal models that allow controlled conditions and experimentation, will need to determine the functions of the long and short isoforms of CAPON and their interaction with other proteins involved in postsynaptic neurotransmission (some of which have also been linked to schizophrenia), and elucidate a possible role for CAPON in psychiatric disorders.

